# Monitoring Immune Responses in IgA Nephropathy: Biomarkers to Guide Management

**DOI:** 10.3389/fimmu.2020.572754

**Published:** 2020-10-06

**Authors:** Haresh Selvaskandan, Sufang Shi, Sara Twaij, Chee Kay Cheung, Jonathan Barratt

**Affiliations:** Mayer IgA Nephropathy Laboratories, Department of Cardiovascular Sciences, University of Leicester, Leicester, United Kingdom

**Keywords:** IgA nephropathy, biomarkers, treatment, Berger’s disease, management biomarkers in IgA nephropathy

## Abstract

IgA nephropathy (IgAN) is the commonest biopsy-reported primary glomerulonephritis worldwide. It has an incidence which peaks among young adults, and 30 to 40% of patients’ progress to end stage kidney disease within twenty years of diagnosis. Ten-year kidney survival rates have been reported to be as low as 35% in some parts of the world. The successful management of IgAN is limited by an incomplete understanding of the pathophysiology of IgAN and a poor understanding of how pathophysiology may vary both from patient to patient and between patient groups, particularly across races. This is compounded by a lack of rigorously designed and delivered clinical trials in IgAN. This is slowly changing, with a number of Phase 2 and 3 clinical trials of novel therapies targeting a number of different putative pathogenic pathways in IgAN due to report in the next 5 years. From our current, albeit limited, understanding of the pathophysiology of IgAN it is unlikely a single therapy will be effective in all patients with IgAN. The successful management of IgAN in the future is, therefore, likely to be reliant on targeted therapies, carefully selected based on an individualized understanding of a patient’s risk of progression and underlying pathophysiology. The potential role of biomarkers to facilitate personalization of prognostication and treatment of IgAN is immense. Here we review the progress made over the past decade in identifying and validating new biomarkers, with a particular focus on those that reflect immunological responses in IgAN.

## Introduction

IgA nephropathy (IgAN) is the most common biopsy reported cause of primary glomerulonephritis worldwide (1–4). It is characterized by IgA deposition in the glomerular mesangium, which variably triggers a series of inflammatory and fibrotic cascades leading to a spectrum of clinical presentations, ranging from asymptomatic non-visible hematuria to rapidly progressive glomerulonephritis (5–7). The incidence of IgAN peaks among young adults and runs a progressive course to end stage kidney disease (ESKD) in up to 40% of patients within twenty years of diagnosis (8, 9). Until recently, predicting which patients were most at risk of progression at the point of diagnosis remained a clinical challenge. The development of the International IgAN Prediction Tool in 2019 has in part addressed this issue, facilitating the provision of timely counseling (10).

Despite this development, there remain two key barriers to the provision of safe, effective care to those diagnosed with IgAN:

1. An incomplete understanding of the pathophysiology of IgAN and a poor understanding of how pathophysiology may vary both from patient to patient and between patient groups, particularly across races.
2. A lack of rigorously designed and delivered clinical trials in IgAN. This is slowly changing, however, with a number of Phase 2 and 3 clinical trials of novel therapies due to report over the next 5 years.

From our current, albeit limited, understanding of the pathophysiology of IgAN it is unlikely a single therapy will be effective in all patients with IgAN. The successful management of IgAN in the future is, therefore, likely to be reliant on targeted therapies, carefully selected based on an individualized understanding of a patient’s risk of progression and underlying pathophysiology. The potential role of biomarkers to facilitate personalization of prognostication and treatment of IgAN is therefore garnering much interest. New biomarkers in IgAN may in the future inform:

1. **Diagnosis:** Kidney biopsy is currently the only diagnostic test of IgAN; however, it is invasive and associated with discomfort, short term restrictions on activity and lifestyle, and some morbidity.
2. **Prognostication:** The International IgAN Prediction Tool provides individualized risk up to 5 years from diagnosis but relies on a number of non-specific markers of kidney damage and can only be used within 6 months of the diagnostic kidney biopsy.
3. **Treatment selection:** At present, kidney biopsy features at time of diagnosis are used by some clinicians to guide some treatment decisions but this approach has not been formally tested or validated outside single centers and is not recommended in international guidelines.
4. **Monitoring response to treatment:** This is currently limited in IgAN to assessment of non-specific measures including urine protein excretion and serum creatinine/estimated glomerular filtration rate (eGFR).

Before any new biomarker is introduced into clinical practice, we must consider whether it is:

1. **Biologically plausible:** the biomarker should be relevant to our understanding of IgAN pathophysiology.
2. **Sensitive:** the biomarker should accurately measure the proportion (ideally 100%) of actual positives that are correctly identified as such (e.g., the percentage of IgAN cases who are correctly identified as having IgAN using the novel biomarker).
3. **Specific:** the biomarker should accurately measure the proportion (ideally 100%) of actual negatives that are correctly identified as such (e.g., the percentage of healthy people who are correctly identified as not having IgAN using the novel biomarker).
4. **Validated:** measurement of the biomarker must be technically validated both within and across laboratories with a single assay system.
5. **Generalizable:** the biomarker should ideally be informative across ethnic groups and geographical regions.
6. **Minimally invasive to collect:** the biomarker should be measurable in easy to collect biological samples such as blood, urine, sputum, and feces.
7. **Relatively resistant to degradation:** the biomarker should be stable under reasonable storage conditions, to allow for the delay between collection of the sample in clinical practice and analysis in a laboratory.
8. **Easy to measure:** the biomarker should be measured using readily available technologies such that most laboratories in the world will have the facilities and expertise to undertake measurement.
9. **Inexpensive:** for rapid integration into clinical pathways the biomarker should be relatively inexpensive to measure using existing technology.

## An Exemplar of Translating Biomarker Discovery From the Bench to the Bedside in IgAN

### The Oxford Classification of IgA Nephropathy

In 2004, the International IgAN Network and the Renal Pathology Society agreed to develop a histopathological scoring system that would “have **clear definitions**, be **simple to use** in clinical practice, be **reproducible** and have a **prognostic value** independent of the clinical parameters at the time of biopsy” (11). Eighteen kidney pathologists, from 10 different countries spanning 4 continents, first undertook extensive iterative work to define pathologic variables with acceptable inter-observer reproducibility. All lesions were identifiable with light microscopy alone, minimizing the requirement for other more expensive and less widely available testing methods. Where groups of such features closely correlated, variables were further selected on the basis of least susceptibility to sampling error and ease of scoring in routine practice. This process identified six pathologic variables that were then used to interrogate prognostic significance independent of clinical data. In a retrospective analysis, sequential clinical data were obtained on 265 adults and children with IgAN from eight countries across four continents who were followed for a median of 5 years. Kidney biopsies from all patients were independently scored for these six lesions by five pathologists blinded to the clinical data. Four lesions, mesangial hypercellularity (M), endocapillary hypercellularity (E), segmental glomerulosclerosis (S), and tubulointerstitial fibrosis/tubular atrophy (T) were finally selected based on simplicity of assessment, independence from other lesions, inter-observer reproducibility and sufficient independence from clinical parameters in their predictive value. Crescents were not included in the initial classification due to a low prevalence, however, following a similar analytical process and separate scoring of 3,096 kidney biopsies the fraction of crescent-containing glomeruli was shown to associate with survival from either a ≥50% decline in eGFR or ESKD adjusting for the covariates used in the original Oxford study and the C score was added to complete the MEST-C score (12, 13). Critical to the translation of the Oxford Classification into routine international clinical practice was the extensive number of independent validation studies, to date, there have been 21 published validation studies, including more than 7,000 patients: 12 Asian, 5 European, 2 North American, and 2 comprising more than one continent (14).

**Figure 1:**
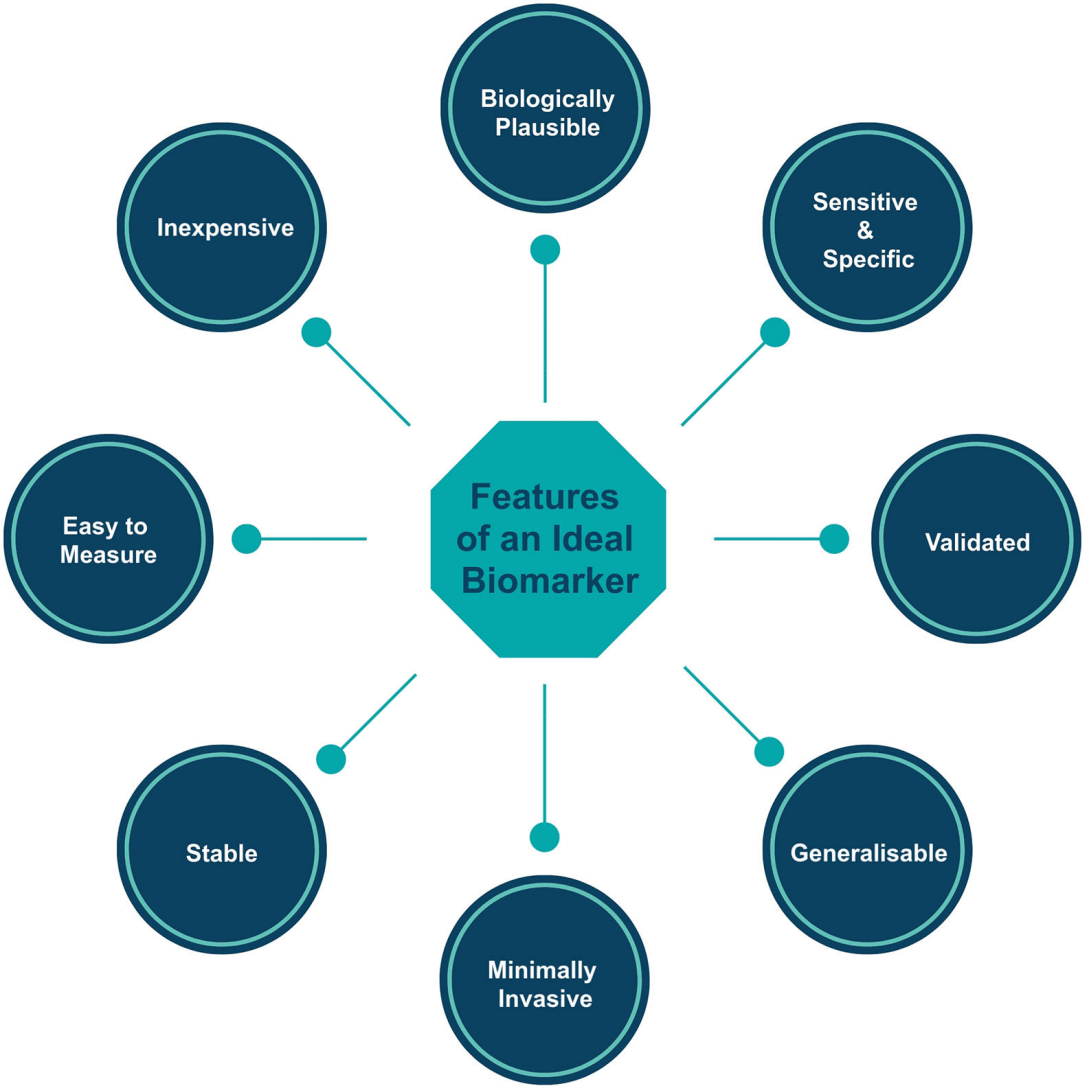
Features of the ideal biomarker.

The Oxford Classification and its component biomarkers fulfil all of the requirements of a good prognostic biomarker set (**Figure 1**) : assessment utilizes an existing stained kidney biopsy section which is universally available in routine clinical care and therefore incurs no additional expense to clinical services, biomarkers were selected based on acceptable inter-observer reproducibility, included cases represented different ages, genders and races to ensure generalizability, and there has been extensive independent cross-continental validation.

As a direct consequence of the international collaboration that delivered the Oxford Classification, research teams from across the world have subsequently worked together to develop the International IgAN Prediction Tool, which for the first time provides patients with an individualized risk of a 50% reduction in eGFR or ESKD (defined as an eGFR < 15 ml/min/1.73 m^2^, transplantation or dialysis) up to 5 years following kidney biopsy. It uses biomarkers that are widely available in all parts of the world, are cheap to measure, are already embedded in routine clinical care and have been individually validated as prognostic biomarkers (**Table 1**). Like the Oxford Classification, the patient population used to derive the tool was ethnically diverse, including 3,067 patients from Europe, China and Japan optimizing generalizability. A further 1564 patients from Europe, Asia, North America, South America, China, and Japan were used to externally validate the model. The tool has recently been validated in a separate study of 1,275 patients and other validation studies will be published in the near future (15).

**Table 1:** The data elements included in the International IgAN Prediction Tool*.

**Estimated GFR at biopsy**	ml/min/1.73m^2^
**Systolic blood pressure**	mmHg
**Diastolic blood pressure**	mmHg
**Proteinuria at biopsy**	g/day
**Age at biopsy**	years
**Race**	
Caucasian	
Chinese	
Japanese	
Other	
**Use of ACE inhibitor or ARB at the time of biopsy**	
No	
Yes	
**MEST M-score**	
0	
1	
**MEST E-score**	
0	
1	
**MEST S-score**	
0	
1	
**MEST T-score**	
0	
1	
2	
**Immunosuppression use at or prior to biopsy**	
No	
Yes	

^*^Using clinical and histologic data at biopsy users can determine a 50% decline in eGFR or kidney failure at selected time intervals. The tool is not validated for use with data obtained remotely from the time of biopsy.

ACE, angiotensin-converting enzyme; ARB, angiotensin receptor blocker; GFR, glomerular filtration rate.

**Figure 2:**
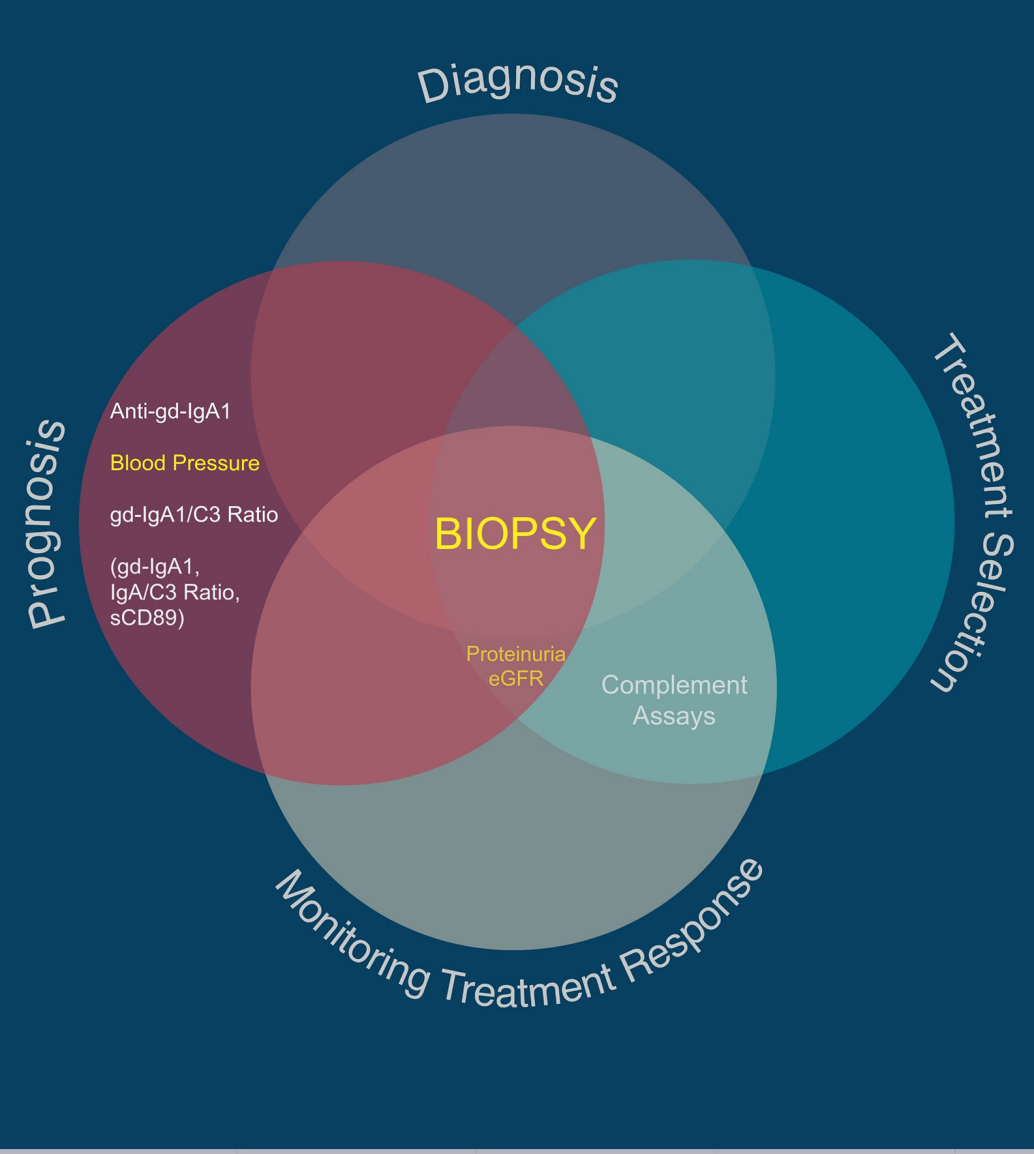
Utility of traditional and suggested utility for novel IgAN-specific biomarkers. Traditional biomarkers are shown in yellow. Proposed novel IgAN-specific biomarkers with the greatest number of associated publications are shown in white, with those in parentheses being regularly reported as useful biomarkers in the literature but with very low quality evidence for sensitivity and specificity in IgAN.

In our view, both the Oxford Classification and the International IgAN Prediction Tool have set the benchmark that all novel biomarkers need to reach to justify translation into clinical practice in IgAN. A large number of biomolecules associated with the immune system have been reported in small discovery studies as candidate biomarkers in IgAN; however, at present none have come close to meeting this benchmark (examples are given in **Tables 2**, **3**) and consequently to date none have entered routine clinical practice. Proposed diagnostic and prognostic biomarkers linked to the immune response in IgAN include immunoglobulin A (IgA) and its associated complexes, components of the complement system, serum, urine and kidney-specific microRNAs and soluble CD89 (76–78). Additional biomarkers have been identified from genome wide association studies, peripheral blood mononuclear cell phenotyping, microbiome profiling, and metabolomic- and proteomic-based interrogation of serum, plasma, urine, and kidney tissue (76). In this review, we have deliberately focussed on those biomarkers that inform clinical decision making in IgAN currently and those most mentioned in the general IgAN literature (**Figure 2**), rather than those biomarkers that might suggest a likely underlying disease mechanism and therefore be potentially useful for drug discovery. We, therefore, discuss the most widely published biomarkers associated with the dysregulated immune response in IgAN, and (i) whether they might inform future clinical care and (ii) how well their performance has been validated and (iii) how likely they are to add value to the traditional biomarkers currently used in clinical practice (**Table 4**).

**Table 2:** Reported putative biomarkers in IgAN (serum, plasma, and whole blood).

Biomarker	Proposed Predictivity Parameter	Sample Size/Controls	Study design	Country of Study	Conclusion	Reference
25-hydroxy-vitamin D	Prognosis	105 IgAN Patients	Prospective cohort study	China	25-hydroxy-vitamin D deficiency was associated with IgAN progression and poorer histological features (MEST-C score)	(16)
Copeptin	Prognosis	59 IgAN patients	Prospective cohort study	Netherlands	Copeptin was associated with disease severity and progression in IgAN (doubling of creatinine, ESKD or commencement of immunosuppressive therapy)	(17)
Fibroblast growth factor 23	Prognosis	180 IgAN patients	Prospective cohort study	Sweden	Serum FGF23 predicted ESKD or >/= 25% reduction in eGFR within 10 years, independent of age, sex, albumin, PTH and bone minerals.	(18)
Matrix Metalloproteinase 7	Prognosis	244 IgAN patients	Retrospective cohort study	China	Elevated MMP7 was independently associated with kidney fibrosis and kidney function decline.	(19)
Neutrophil to lymphocyte ratio	Response to treatment	66 IgAN patients in remission, 33 persistent disease activity	Retrospective cohort study	China	Patients with an NLR of <2.43 were more likely to achieve remission with corticosteroids.	(20)
NGAL (urine and serum)	Prognosis	121 IgAN patients	Prospective cohort study	Korea	Elevated Urine and serum NGAL combined was associated with a greater decline in eGFR.	(21)
Oxylipins and arachidonic acid metabolites	Response to treatment	96 IgAN patients	Randomized, placebo controlled, double blind clinical trial	United States of America	Oxylipins and arachidonic acid metabolites changes may predict remission in response to fish oil in IgAN	(22)
Platelet derived growth factor	Diagnosis	33 IgAN patients, 48 disease controls	Prospective case control study	Germany	PDGF were elevated in patients with IgAN compared to disease controls	(23)
Plasma CRP	Prognosis	174 IgAN patients	Retrospective cohort study	Finland	CRP, albumin and white blood cell count elevations are associated with IgAN progression	(24, 25)
Plasma albumin						
White blood cell count						
Plasma insulin level						
Plasma alpha defensins	Diagnosis	169 IgAN patients, 83 healthy controls	Retrospective case control study	China	Plasma alpha defensins are higher in IgAN patients compared to healthy controls.	(26)
Plasma acylcarnitines	Prognosis	81 IgAN patients	Retrospective cohort study	China	Elevated plasma acylcarnitines were associated with IgAN progression	(27)
Soluble ectodomain of vascular cell adhesion marker 1	Diagnosis and Prognosis	327 IgAN patients, 55 healthy controls	Retrospective case control study	China	sVCAM-1 is elevated in IgAN patients compared to healthy controls and was associated with proteinuria, low eGFR and histological severity (MEST-C score)	(28)
Soluble interleukin 2 receptor alfa	Prognosis	194 IgAN patients, 84 healthy controls	Prospective case control study	Sweden	sIL-2Ra predicted progression of kidney disease in IgAN patients.	(29)
Soluble ST2	Diagnosis and Prognosis	74 IgAN patients, 34 healthy controls	Prospective case control study	China	Soluble ST2 was higher in IgAN patients, and correlated positively with worsening histology (WHO histological classification), negatively with eGFR and positively with proteinuria.	(30)
Transforming growth factor ß1	Diagnosis and Prognosis	100 IgAN patients, 56 health controls.	Retrospective case control study	China	TGFß1 was elevated in patients with IgAN compared to healthy controls, and was more elevated in patients with more severe histological lesions (WHO classification)	(31)
Tumour Necrosis Factor ß	Diagnosis and Prognosis	147 IgAN patients, 126 healthy controls	Retrospective case control study	China	TNFa is higher in patients with IgAN compared to healthy controls, and positively correlated with proteinuria and histological severity. TNFa negatively correlated with eGFR.	(32)
Tumour Necrosis Factor receptor	Prognosis	347 IgAN patients	Prospective cohort study	Korea	Elevated TNFR concentrations were associated with IgAN histological severity (local grading system) and disease progression	(33)
	Prognosis	106 IgAN patients, 34 healthy controls	Retrospective case control study	Japan	TNFR correlated with urinary protein creatinine ratio and degree of interstitial fibrosis	(34)
Uric Acid	Prognosis	623 IgAN patients	Retrospective cohort study	China	Uric acid levels correlated with severity of tubulointerstitial damage (MEST-C score and Beijing classification system).	(35)
	Prognosis	226 IgAN patients	Prospective cohort study	Japan	Elevated uric acid is a risk factor of IgAN progression.	(36)
	Prognosis	611 IgAN patients	Retrospective cohort study	Japan	Elevated uric acid is a risk factor of IgAN progression, but only in those with CKD3a	(37)
	Prognosis	202 IgAN patients	Retrospective cohort study	Finland	Uric acid levels correlated with severity of tubulointerstitial damage (Local grading system).	(38)
	Prognosis	111 IgAN patients	Prospective cohort study	Turkey	Elevated uric acid is a risk factor of IgAN progression.	(39)
	Prognosis	93 IgAN patients	Retrospective cohort study	Turkey	Elevated uric acid is a risk factor of IgAN progression.	(40)

**Table 3:** Reported putative biomarkers in IgAN (urine).

Biomarker	Proposed Predictivity Parameter	Sample Size/Controls	Study design	Country of Study	Conclusion	Reference
Adiponectin	Diagnosis and Prognosis	12 IgAN patients, 10 disease controls and 24 healthy controls	Retrospective case control study	Japan	Adiponectin correlated with urine albumin creatine ratio in both IgAN patients and disease controls.	(41)
α-1-microglobulin	Diagnosis	17 IgAN patients, 16 disease controls, 10 healthy volunteers	Retrospective case control study	Japan	Urinary α-1-microglobulin was lower in patients with IgAN compared to healthy volunteers and disease controls.	(42)
Angiotensinogen	Prognosis	36 IgAN patients, 14 disease controls and 15 healthy controls	Retrospective case control study	Korea	Urinary angiotensinogen correlated with urinary protein creatine ratio, but was not specific for IgAN.	(43)
Aquaporin 2	Diagnosis	44 IgAN patients, 21 disease controls, 40 healthy controls	Retrospective case control study	Italy	Urinary aquaporin 2 was higher in patients with IgAN compared to disease and healthy controls. Higher aquaporin 2 levels were associated with proteinuria and hypertension.	(44)
β−2-microglobulin	Prognosis	51 IgAN patients	Retrospective cohort study	Korea	β−2-microglobulin levels correlated with kidney function, proteinuria and was a predictor of disease progression	(45)
Calprotectin & TIMP2*IGFBP7	Prognosis	113 IgAN patients	Prospective cohort study	Germany	Calprotectin and TIMP2*IGFBP7 were not useful in discriminating between patients at risk of progressive disease and those who or not, or those who achieved remission and those who did not.	(46)
CD89-Transglutaminase-2 Product	Prognosis	160 IgAN/HSP patients	Prospective cohort study	Belgium, Poland, Italy	CD89 -Transglutaminase product was associated with proteinuria in IgAN and HSP patients	(47)
C-megalin	Prognosis	73 IgAN patients, 5 disease controls	Retrospective case control/cohort study	Japan	C-megalin was associated with mesangial hypercellularity and chronic extra capillary abnormalities in IgAN patients.	(48)
Collagen type III neo-epitope fragment (C3M)	Prognosis	48 IgAN patients	Prospective cohort study	Greece	Urine C3M was lower in IgAN patients who subsequently developed progressive kidney disease	(49)
Collagen type 4	Prognosis	34 IgAN patients	Prospective cohort study	Japan	Elevated urinary collagen 4 was associated with histologically severe lesions on biopsy	(50)
CXCL1	Prognosis	425 IgAN patients, 160 disease controls and 74 healthy controls	Retrospective case control study	China	Urinary CXCL1 correlated with proteinuria, tubular atrophy and interstitial fibrosis and was independently associated with a greater risk of kidney function decline.	(51)
Epidermal growth factor	Prognosis	33 IgAN Patients	Retrospective cohort study	Greece	EFG negatively correlated with the extent of fibrosis in kidney biopsy	(52)
	Prognosis	58 IgAN patients	Retrospective cohort study	Spain		(53)
Epidermal growth factor to monocyte chemotactic peptide 1 ratio	Prognosis	132 IgAN patients	Prospective cohort study	Italy	A low EGF:MCP1 ratio was associated with a greater risk of kidney function decline, and was found to be an independent risk factor for kidney function decline.	(54)
Exosomes	Diagnosis and Prognosis	55 IgAN patients, 24 healthy controls, 25 disease controls	Retrospective case control study	China	Urinary exosomes were compared to healthy and disease controls, and correlated with histological severity.	(55)
Exosomal CCL2 mRNA	Diagnosis and Prognosis				Exosomal CCL2 was compared to healthy and disease controls, and correlated with tubulointerstitial inflammation and kidney function deterioration	
Free kappa light chains	Diagnosis and Prognosis	49 IgAN patients, 42 disease controls, 40 healthy controls	Retrospective case control study	Italy	Free kappa light chains were reduced in IgAN compared to healthy and disease controls. Concentration was inversely correlated with histological severity (MEST-C)	(56)
IgA-Uromodulin complex	Diagnosis	126 IgAN patients, 94 disease controls	Retrospective case control study	Japan	Urinary IgAN-Uromodulin complex had a sensitivity 81.7%, specificity of 73.4% and a diagnosis efficiency of 78.2% for IgAN	(57)
IL1β	Diagnosis	13 IgAN, 3 Henoch-Schönlein purpura, 11 disease controls, 5 healthy controls	Retrospective case control study	China	IL1 β was elevated in the urine if IgAN patients compared to healthy and disease controls	(58)
IL6	Prognosis	33 IgAN Patients	Retrospective cohort study	Greece	IL6 was significantly elevated in IgAN patients with more kidney fibrosis. IL6 was also shown to be associated with histological progression in a Japanese cohort.	(52)
		58 IgAN patients	Retrospective cohort study	Spain		(53, 59)
Kidney injury molecule 1	Prognosis	113 IgAN patients	Prospective cohort study	Germany	KIM1 was not useful in discriminating between patients at risk of progressive disease and those who or not, or those who achieved remission and those who did not.	(46)
	Response to Treatment	37 IgAN patients	Retrospective cohort study	Korea	KIM1 was reduced in response to treatment, which included immunosuppression. The study was not set up to predict which patients would respond to treatment	(60)
	Prognosis	51 IgAN patients	Retrospective cohort study	China	KIM1 correlated with severity of tubulointerstitial fibrosis on biopsy (MEST-C sore).	(61)
	Diagnosis and Prognosis	202 IgAN patients, 46 disease controls, 60 healthy controls	Retrospective case control study	China	KIM1 was elevated in IgAN patients compared to disease and healthy controls, and correlated with proteinuria, creatinine and tubulointerstitial injury. KIM 1 predicted kidney function decline	(62)
	Diagnosis and Prognosis	40 IgAN patients, 10 healthy controls	Retrospective case control study	Korea	KIM1 was elevated in IgAN patients compared to healthy controls and correlated with histological severity (Lee’s grades).	(63)
	Prognosis	65 IgAN patients, 65 healthy controls	Retrospective case control study	Netherlands	KIM1 correlated with degree of proteinuria and predicted kidney function decline	(64)
Laminin G like 3	Diagnosis	43 IgAN patients, 65 disease controls, 30 healthy controls	Retrospective case control study	France	Laminin G like 3 was reduced compared to healthy and disease controls, but only in those with conserved kidney function.	(65)
	Diagnosis and Prognosis	49 IgAN patients, 42 disease controls, 40 healthy controls	Retrospective case control study	Italy	Laminin G like 3 was reduced in IgAN compared to healthy and disease controls. Concentration was inversely correlated with histological severity (MEST-C)	(56)
Low molecular weight proteins	Prognosis	70 IgAN patients	Prospective cohort study	Netherlands	Urinary low molecular weight proteins offered no benefit over traditional risk factors in a multivariate model.	(66)
Mannose binding lectin	Prognosis	162 IgAN patients, 50 healthy controls	Retrospective case control study	China	Urinary MBL correlated with proteinuria, creatinine, blood pressure, histological severity and predicted kidney disease progression.	(67)
Matrix metalloproteinase 7	Prognosis	946 IgAN patients	Prospective cohort study	China	MMP7 was an independent risk factor for disease progression, and improved the risk prediction of the MEST-C score	(68)
MCP1	Prognosis	33 IgAN Patients	Retrospective cohort study	Greece	MCP1 was elevated in IgAN patients with more kidney fibrosis.	(52)
		58 IgAN patients	Retrospective cohort study	Spain		(53)
Neutrophil Gelatinase associated lipocalin	Prognosis	121 IgAN patients	Retrospective cohort study	Korea	Elevated urinary NGAL predicts kidney disease progression.	(21)
	Diagnosis and Prognosis	70 IgAN patients, 40 healthy controls	Retrospective case control study	China	NGAL was elevated in patients with IgAN compared to healthy controls, and correlated with histological severity (Lee’s Grades)	(69)
	Prognosis	113 IgAN patients	Prospective cohort study	Germany	NGAL was not useful in discriminating between patients at risk of progressive disease and those who or not, or those who achieved remission and those who did not.	(46)
Periostin	Prognosis	345 IgAN patients, 56 disease controls	Prospective cohort study	Korea	Urinary periostin/creatinine values were elevated in patients with fibrosis and tubular atrophy, and in those who developed progressive kidney disease.	(70)
Podocalyxin	Prognosis	51 IgAN patients	Retrospective cohort study	Japan	Podocalyxin and podocyte count correlated with histological severity (Shigematsu classification)	(71)
Podocytes						
serum-and-glucocorticoid inducible kinase 1	Diagnosis and Prognosis	76 IgAN patients, 33 healthy volunteers	Retrospective case control study	China	Urinary SGK1 was elevated in patients with IgAN compared to healthy controls, correlated with the degree of tubulointerstitial damage in IgAN (MEST-C score), proteinuria and kidney insufficiency	(72)
Soluble transferrin receptor	Diagnosis and Prognosis	71 IgAN/HSP patients, 61 disease controls	Retrospective case control study	Belgium	soluble transferrin receptor concentrations were higher compared to disease controls, and correlated with proteinuria	(73)
TGFβ	Prognosis	58 IgAN patients	Retrospective cohort study	Spain	TGFβ was elevated in IgAN patients with more kidney fibrosis.	(53)
TNFα	Diagnosis	13 IgAN, 3 Henoch-Schönlein purpura, 11 disease controls, 5 healthy controls	Retrospective case control study	China	TNFα was elevated in the urine if IgAN patients compared to healthy and disease controls	(58)
Uromodulin (fragment)	Diagnosis	32 IgAN patients, 36 disease controls, 30 healthy controls	Prospective case control study	China	Uromodulin (fragment) distinguished IgAN patients from disease and healthy controls.	(74)
Vitamin D Binding Protein	Response to Treatment	80 IgAN patients	Prospective cohort study	China	Urinary vitamin D binding proteins were elevated in IgAN patients whose proteinuria failed to settle with irbesartan.	(75)

**Table 4:** Comparison of the clinical utility of traditional and proposed novel IgAN-specific biomarkers.

**Traditional Biomarkers**								
**Biomarker**	**Affordable and accessible**	**Minimally Invasive**	**Biologically Plausible**	**Diagnostic**	**Prognostic**	**Treatment Selection**	**Monitor Treatment Response**	**Widely Validated**
Proteinuria								
Hypertension								
eGFR								
Histology								
**Proposed Novel Biomarkers in blood and urine**								
serum IgA								
IgA/C3								
Polymeric IgA								
Secretory IgA								
Galactose-deficient IgA1								
Urinary galactose-deficient IgA1								
GalNAc content of IgA1 hinge region								
Anti-Gd-IgA1								
Anti-Gd-IgA1 – Gd-IgA1 complexes								
Soluble CD89								
Complement Assays								
microRNAs								
PBMC Phenotyping								

Green: sufficient evidence present to justify use; Yellow: Some evidence present to justify use; Red: Insufficient evidence present to justify use.

## Traditional Biomarkers Currently Used in the Management of IgAN

Diagnosis and prognostication in IgAN have changed little over the past few decades. A kidney biopsy remains the only way to diagnose IgAN. Current strategies for predicting the likelihood of progression in IgAN and defining an “at risk” population suitable for a trial of immunomodulating therapy are based on a panel of biomarkers collected as part of routine clinical practice that combine non-IgAN specific measures [proteinuria, blood pressure (BP), eGFR], and IgAN-related measures from the diagnostic kidney biopsy. Proteinuria, eGFR, BP, and histopathological features of the kidney biopsy are by far the most valuable biomarkers we currently have in IgAN. All have been thoroughly and independently validated in cohorts from across the globe and provide unrivalled diagnostic and prognostic information. Any new diagnostic or prognostic biomarker must show additional value above these measures (**Figure 1**), and to date none have achieved this.

### Kidney Biopsy and Histomorphometry

#### Diagnosis

Since the initial description in 1968, a kidney biopsy demonstrating dominant or co-dominant mesangial IgA deposition is the only way to diagnose IgAN (79).

#### Prognosis

As already discussed, the Oxford Classification defines five histopathological lesions, MEST-C, that independently predict prognosis in IgAN, has been adopted as the international standard for kidney biopsy reporting in IgAN and forms a key component of the International IgAN Prediction Tool (10). Furthermore, there is compelling experimental evidence that each of these lesions associates with known pathogenic pathways triggered by mesangial deposition of IgA immune complexes in IgAN (80).

#### Treatment Selection

Currently, there are no validated features in the kidney biopsy that can be used to guide treatment selection in IgAN, including the MEST-C score. Retrospective and uncontrolled clinical trial data have suggested that endocapillary hypercellularity, a measure of glomerular macrophage accumulation, may be modifiable with systemic immunosuppression, leading to an improved outcome in Chinese and Caucasian patients (13, 81, 82). However, there have been no studies that have randomized patients on the basis of this histopathological feature and therefore international guidelines do not currently recommend basing treatment decisions on kidney biopsy features.

#### Monitoring Response to Treatment

Repeat kidney biopsy is rarely performed in IgAN, however, over the past 5 years there have been a few small repeat kidney biopsy studies published, both single center retrospective cohort studies and prospective clinical studies of immunosuppression (81, 82). At present, however, there is too little data to draw any firm conclusions on the value of repeat biopsy in IgAN.

Despite offering diagnostic, prognostic and possibly treatment relevant information the kidney biopsy is invasive and associated with discomfort, short term restrictions on activity and lifestyle, and appreciable morbidity. Furthermore, due to sampling error, kidney biopsies can provide misleading information and occasionally be uninformative.

### Proteinuria

#### Prognosis

The presence and the amount of proteinuria have long been recognized as risk factors for kidney function decline in kidney disease (83), and proteinuria is an important prognostic biomarker in IgAN (84, 85). In epidemiological studies time averaged proteinuria (TAP) is the strongest independent predictor of IgAN progression and ESKD. The 10-year risk of ESKD is only 5% if TAP is <1 g/day, increasing to 60% with TAP >3 g/day (86). This finding has been validated in three large studies in Korea (Islan Hospital, 500 patients) Europe (VALIGA, 1,147 patients), and China (Nanjing Registry, 1,155 patients), the latter two retrospective studies also demonstrated a kidney survival benefit in those maintaining TAP <0.5 g/day (87–89). Proteinuria is a key component of the International IgAN Prediction Tool.

#### Treatment Selection

Persistent, elevated proteinuria >1 g/24 h, despite maximal supportive care, is currently used to define an “at risk” IgAN population whose risk of progression is sufficiently high to warrant a trial of immunosuppression, with the attendant risk of side effects. Likewise, persistent proteinuria >1 g/24 h is also used as an entry criteria for all currently recruiting Phase 2 and 3 trials of immunomodulatory therapies in IgAN.

#### Monitoring Response to Treatment

An early reduction in proteinuria with a range of interventions has been associated with improved long term kidney survival in IgAN (86, 90). In 2019, the FDA (Food and Drug Administration) accepted an early change in proteinuria as a reasonably likely surrogate end point for a treatment’s effect on progression to ESKD in IgAN. An early change in proteinuria is now being used to monitor the response to a range of novel and repurposed therapies currently being evaluated in IgAN (NCT03841448, NCT03762850, and NCT01738035).

Measurement of proteinuria offers a non-invasive and inexpensive way to risk stratify patients with IgAN and monitor the response to new therapies. There is extensive evidence that proteinuria is not only a biomarker of glomerular injury but also a contributor to downstream kidney inflammation through a number of established mechanisms, including tubular chemokine and complement activation (91, 92) and therefore is a biologically plausible biomarker for risk of progression. A major challenge, however, is that proteinuria can vary within an individual and is affected by exercise, dietary sodium excretion and the method of assessment. The gold standard in clinical trials is to measure a protein-to-creatinine ratio (UPCR) on an aliquot of a 24-h urine collection. For most in routine clinical practice, a 24-h urine collection is impractical and instead either a first morning or randomly timed urine is used to measure a UPCR, both of which are prone to significant variability (93).

While albuminuria and microalbuminuria offer value as an early biomarker of disease progression in other kidney conditions, there is at present minimal data to support albuminuria as a superior biomarker to proteinuria in IgAN (94, 95). Microalbuminuria has been shown to co-present with hematuria more often in those with IgAN; however, its sensitivity and specificity are too low to be of any value in clinical practice (96). Furthermore, the albumin-to-creatine ratio (ACR) yields little benefit over UPCR in IgAN (97, 98).

### Blood Pressure

#### Prognosis

Hypertension at diagnosis is an established risk factor for kidney function loss in all forms of kidney disease, including IgAN (10, 84, 86, 99). The BP at diagnosis is included in the International IgAN Prediction Tool. BP control improves outcomes at 20 years, with the incidence of dialysis or death in a cohort of French patients being 5% for normotensives, 19% for controlled hypertensives (≤130/80 mmHg), and 42% in uncontrolled hypertensives (100).

BP measurement is a non-invasive, inexpensive, and extensively validated prognostic biomarker in IgAN.

### Estimated Glomerular Filtration Rate

#### Prognosis

The eGFR at time of diagnosis is a well-established predictor of future risk of kidney function decline and ESKD in IgAN. In a study of 2,269 Japanese patients, the incidence of ESKD was 90% in those who presented with a serum creatinine ≥ 220 μmol/L (101), and similar findings have been reported in multi-ethnic cohorts from the USA, Europe and Asia (100, 102–105). eGFR is included in the International IgAN Prediction Tool.

#### Treatment Selection

The current KDIGO Clinical Practice Guidelines for Glomerulonephritis suggest that immunomodulatory therapy should not be used in patients with an eGFR < 5 0 ml/min/1.73 m^2^, and all currently recruiting clinical trials in IgAN have a lower level of eGFR (between 30 and 45 ml/min/1.73 m^2^) below which patients are excluded from the study. This is for two principal reasons. Firstly, at lower eGFR levels it is predicted there will be less immunological activity and more fibrotic remodeling in the kidneys which means the disease will be unresponsive to immunosuppression. Secondly, at lower levels of eGFR the risk of treatment emergent adverse events increases and, therefore, the risk to benefit ratio becomes unacceptably high.

#### Monitoring Response to Treatment

Fundamentally, any therapy for IgAN should preserve eGFR. Measuring meaningful changes in eGFR in a slowly progressive disease such as IgAN has, however, presented significant challenges to researchers and regulators and, until recently, severely impacted on the attractiveness of studying new therapies in IgAN. The rate of eGFR decline, or eGFR slope, has been proposed as a valid end point in clinical trials to facilitate the recruitment of early disease stage patients, in whom treatment benefits may otherwise be either missed or would require costly, prolonged studies to capture end points seen in late stage kidney disease (106–108). Two- and three-year eGFR-based end points are now being used in all currently recruiting phase 3 studies of immunotherapies in IgAN to confirm response to treatment.

While proteinuria, BP, and eGFR are all minimally invasive, easily measurable, biologically plausible, relatively inexpensive and extensively validated, they each have their own limitations and all are non-specific and, therefore, tell us nothing about the pathophysiology operating in an individual. In order to be adopted into clinical practice any new IgAN-specific biomarker must, at the very least, provide insights into patient specific disease mechanisms to enable a tailored approach to treatment selection, which at present traditional biomarkers cannot deliver.

## Proposed Novel Biomarkers That May Help in Monitoring the Immune Response in IgAN

### Immunoglobulin A

#### Biological Plausibility

As the diagnosis of IgAN is based on the presence of mesangial IgA deposition it is not surprising that IgA was one of the first potential biomarkers studied in IgAN.

#### Diagnosis

Total serum IgA is elevated in approximately 50% of all cases of IgAN, however, this is neither sensitive or specific enough to be diagnostic of IgAN (109). Van der Boog et al. demonstrated that levels of the high molecular weight fraction of serum IgA, polymeric IgA (containing the circulating IgA-immune complexes), were elevated in 51 Dutch patients with IgAN compared to healthy subjects, but again sensitivity and specificity were insufficient to be of diagnostic value in IgAN (110). Secretory IgA (sIgA) is the major immunoglobulin secreted at mucosal surfaces but can also be found in the serum. Levels of sIgA are elevated in a proportion of patients with IgAN compared to healthy subjects (111, 112), but again sIgA is not sensitive or specific for IgAN (levels are also raised in other disease states including other primary glomerulonephritides and gut disorders) (111, 113).

In an effort to enhance the diagnostic power of serum IgA levels some investigators have promoted the potential value of measuring the serum IgA/C3 ratio in IgAN. Tomino et al. reported that the IgA/C3 ratio was significantly elevated in Japanese IgAN patients compared to disease controls (114–116), and this has been reproduced in separate Japanese and Chinese IgAN cohorts (117, 118). These observations have not, however, been confirmed in Caucasian cohorts and while the IgA/C3 ratio is elevated in IgAN it still does meet the sensitivity and specificity for a diagnostic test.

#### Prognosis, Treatment Selection, and Monitoring Response to Treatment

A small number of studies have examined the relationship of total serum IgA levels, serum IgA/C3 ratio, serum levels of polymeric IgA, and levels of urinary sIgA with clinical disease parameters (hematuria, proteinuria, eGFR, and severity of histopathological injury) and found inconsistent associations (110). Tan et al. measured urinary sIgA in 202 Chinese patients and found higher sIgA concentrations in patients with more severe histopathological findings, defined as Haas-IV or V lesions (119). However, these patients also had higher serum creatinine levels, higher BPs and higher levels of proteinuria. There seemed to be little additive value of measuring urinary sIgA concentrations over traditional biomarkers (120).

## The Four Hit Hypothesis of IgAN

**Figure 3:**
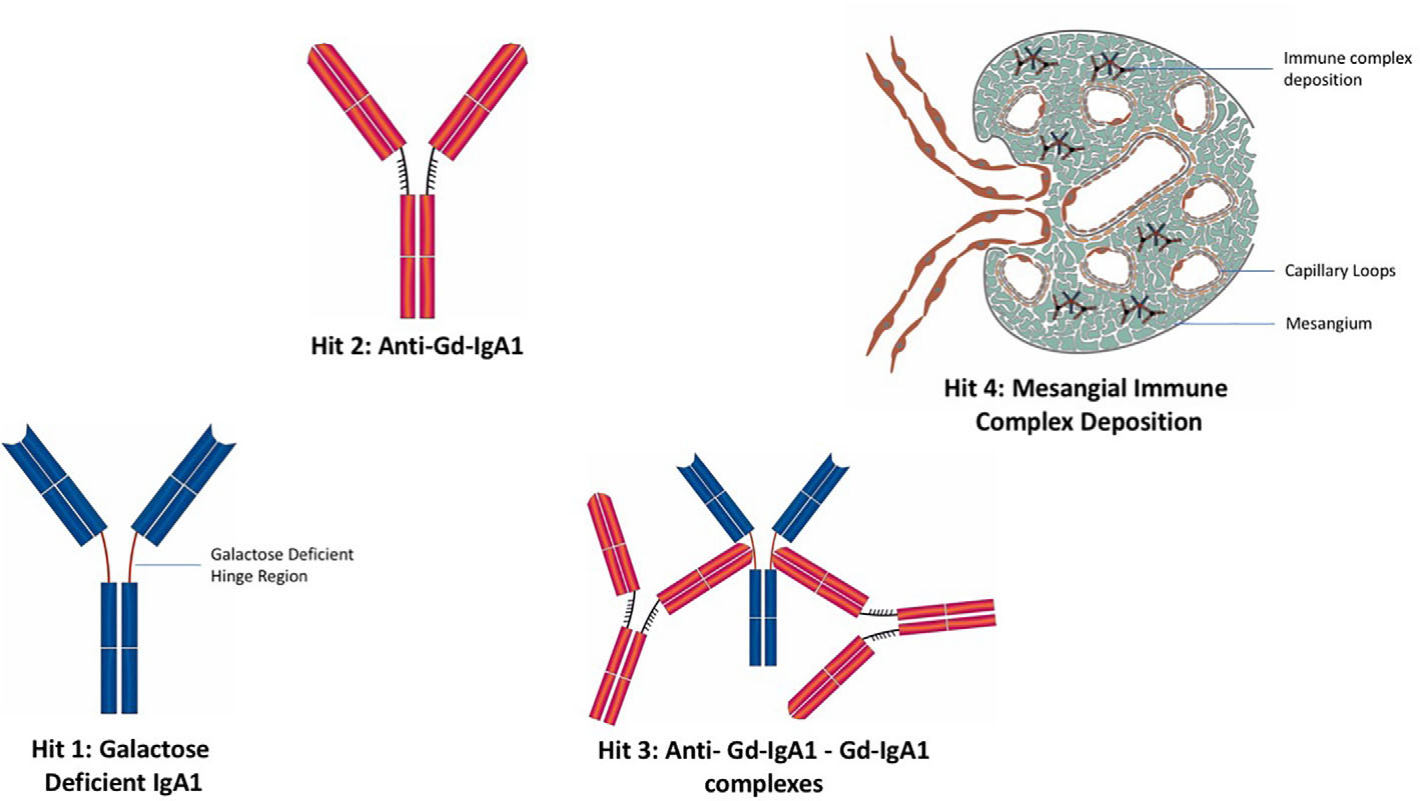
The “four hit” hypothesis of IgA nephropathy. Hit 1: Appearance in the circulation of increased levels poorly *O*-galactosylated IgA1 (gd-IgA1). Hit 2: Generation of IgG and IgA autoantibodies directed against gd-IgA1. Hit 3: Formation of anti-gd-IgA1-gd-IgA1 immune complexes. Hit 4: Deposition of IgA immune complexes in the glomerular mesangium and consequent development of inflammatory and fibrotic processes in the kidney.

A widely reported paradigm to explain the pathophysiology of IgAN is the “four hit” hypothesis, which postulates four separate pathophysiological processes are needed in order for IgAN to develop (**Figure 3**) (121). The first hit is the appearance in the circulation of increased levels of poorly *O*-galactosylated IgA1 (gd-IgA1). This IgA1 may also exhibit reduced *O*-linked sialylation and a reduction in *N*-acetygalactosamine (GalNAc) residues at the hinge region of IgA1 and is believed to be derived from mucosally primed plasma cells (122). The second hit is the generation of IgG and/or IgA autoantibodies directed against the poorly *O*-galactosylated hinge region of gd-IgA1, with the third hit being the formation of anti-gd-IgA1-gd-IgA1 immune complexes. The fourth and final hit is the variable development of inflammatory and fibrotic processes in the kidney, triggered by the deposition of anti-gd-IgA1-gd-IgA1 immune complexes in the mesangium (121). We will deal with each of these hits and their potential to act as biomarkers of the immune response in IgAN.

### Galactose-Deficient IgA1 (HIT 1)

#### Biological Plausibility

The generation of gd-IgA1 is widely considered to be the first hit of the “four hit” hypothesis in IgAN (**Figure 3**), and its potential as a biomarker has been explored by a number of groups.

#### Diagnosis

Serum levels of gd-IgA1 have been reported as elevated, compared to healthy subjects and kidney disease controls, in multiple IgAN cohorts from across the globe including cohorts from Japan, Italy, China, UK, and US (123–127). Similarly, urinary gd-IgA1 has been reported to be elevated in IgAN, compared to disease controls and healthy subjects and also to correlate with proteinuria (128). Despite these associations, gd-IgA1 has no current value as a diagnostic test of IgAN as gd-IgA1 levels lack sensitivity due to the highly variable levels within IgAN cohorts, making a standardized threshold for diagnosis impossible, particularly as there is no standardized and accepted method to measure gd-IgA1 (125). Perhaps, more importantly, gd-IgA1 lacks specificity for IgAN, most notably, gd-IgA1 serum levels are equally elevated in unaffected relatives of IgAN cases (129–131).

#### Prognosis

Zhao et al. reported an association between higher serum gd-IgA1 levels and a greater risk of progressive kidney disease, defined as 50% decline in eGFR, ESKD or death, in a Chinese cohort with a median follow up of 4 years. This association remained after adjustment for immunosuppression use, angiotensin converting enzyme inhibitor use, proteinuria, histopathological severity (based on the Haas classification), and eGFR at the time of presentation (132). However, at least two other groups have found no association between serum gd-IgA1 levels and disease severity (124, 133). Indeed, a meta-analysis of 22 studies (1,657 patients) found no association between serum gd-IgA1 levels and IgAN severity, suggesting that the serum gd-IgA1 level offers little in terms of a prognostic biomarker at present (134).

More recently, the plasma gd-IgA1/C3 ratio has been reported as an independent risk factor for IgAN progression in a large prospective study of 1,210 Chinese patients (135). There is no similar data available for Caucasian patients. We do not know yet whether addition of the plasma gd-IgA1/C3 ratio will improve the prognostic performance of the International IgAN Prediction Tool and this needs to be formally tested.

#### Treatment Selection and Monitoring Response to Treatment

Similarly, there is little information to support the use of serum gd-IgA1 levels in guiding treatment choices. There have been a small number of studies from Japan reporting a reduction in the serum levels of gd-IgA1 following tonsillectomy and pulsed steroid treatment, and that this reduction is associated with an improved kidney outcome (136, 137). Iwatani et al. reported a small case series of adult Japanese IgAN cases (n = 7, four demonstrating remission) where the GalNAc content of serum IgA1 hinge region glycopeptides was marginally increased following tonsillectomy and pulsed steroid treatment, and this increase correlated with remission (138). However, the major challenge interpreting such data based on treatment effects of tonsillectomy and pulsed steroid therapy is that there remains significant controversy over whether this regimen actually alters IgAN outcome.

In addition to measuring serum levels of gd-IgA1, and the “Hit 2”autoantibodies discussed below, a number of investigators have examined peripheral blood lymphocyte and mononuclear cell subsets in an effort to identify changes in the key immune cells responsible for gd-IgA1and autoantibody synthesis, as well as those mediating inflammation in IgAN (139–143). Results of these studies are inconsistent and none have been validated and therefore their utility as biomarkers in IgAN is uncertain. Observations in IgAN include a higher frequency of circulating CXCR5^+^CD4^+^ T cells (127), expansion of the non-classical monocyte subset (144), increase in the number of CD23^+^ and CD19^+^CD5^+^ B-cells, and increased expression of CD62L by lymphocytes (145–148). In a study by Cox et al., a microarray analysis of peripheral blood mononuclear cells (PBMC) identified dysregulation in the PI2K/Akt and WNT-beta-catenin signaling pathways along with dysregulation in apoptotic pathways (142). A single study has examined PBMC in urine in IgAN and this demonstrated a correlation between CD14^+^ and CD56^+^ cells in the urine and the presence of crescents on biopsy (149). In summary, results of peripheral blood immunophenotyping by flow cytometry have been disappointing and have failed to identify consistent changes in IgAN that could be used as viable clinical biomarkers, although it is fair to say that the studies performed thus far are limited in scope and numbers. It is highly likely that immunophenotyping will be extensively re-visited with the commencement of a number of Phase 2 clinical trials of B cell directed therapies targeting BAFF {B-cell activating factor [BLyS (B-lymphocyte stimulator)]} and APRIL (A PRoliferation-inducing ligand), two factors critical for T cell independent IgA class switch recombination and B cell proliferation and survival.

### Autoantibodies Against gd-IgA1 (HIT 2)

#### Biological Plausibility

While generation of autoantibodies against gd-IgA1 constitutes the second hit of the “four hit hypothesis” (**Figure 3**), there remains significant debate over the absolute requirement for IgG (and IgA) autoantibody production in IgAN. In many IgAN kidney biopsy series, a significant number of cases do not demonstrate mesangial IgG, classical pathway complement activation is rare and drugs commonly and effectively employed in autoimmune diseases such as rituximab, cyclophosphamide, and mycophenolate mofetil have been shown to be ineffective in IgAN. It seems more likely that IgA-immune complexes form through direct interaction of polymeric IgA molecules, while the development of autoantibodies, when they occur, may amplify immune complex formation but are not an absolute requirement for immune complex formation in IgAN.

#### Diagnosis

Yanagawa et al. found that gd-IgA1 specific IgG and IgA levels were elevated in a Japanese IgAN cohort compared to disease controls and healthy subjects (123). Similar findings were reported by Berthoux et al. in a French cohort (150). However, up to 25% of non-IgAN CKD patients also have elevated levels of these autoantibodies meaning that they are not suitable to be used as a diagnostic test in IgAN, even when combined with serum levels of gd-IgA1 (123).

#### Prognosis

In a study by Berthoux et al. looking at outcomes in French IgAN cohort serum levels of gd-IgA1–specific IgG and IgA autoantibodies were associated with the risk of disease progression (150). It is not known whether inclusion of these autoantibody levels will improve the prognostic performance of the International IgAN Prediction Tool and this needs to be formally tested before any recommendation can be made concerning their prognostic value in IgAN.

#### Treatment Selection and Monitoring Response to Treatment

One might imagine that if IgAN were a classical autoimmune disease that immunosuppression would reduce autoantibody levels and induce remission of disease. This is not the case in IgAN. As an example, rituximab treatment is associated with peripheral CD20 depletion, reduction in autoantibody levels (PLA2R, PR3- and MPO ANCA, and ds-DNA) and clinical remission in a number of kidney autoimmune diseases (151–153). By contrast, in IgAN rituximab treatment is associated with a similar level of CD20 depletion but has no measurable effect on serum gd-IgA1 or IgG autoantibody levels (154). This difference in response in IgAN suggests that the simple measurement of the putative autoantigen (gd-IgA1) and autoantibody may well not be sufficient to monitor response to therapy in IgAN, or indeed inform therapeutic options, as conventional immunotherapies successfully employed to treat autoimmune disease (often based on the presence of an autoantibody) have shown very little efficacy in IgAN.

### Anti-gd-IgA1–gd-IgA1 Immune Complexes (HIT 3)

Anti-gd-IgA1–gd-IgA1 complexes constitute the third hit of the “four hit” hypothesis (**Figure 3**). In a small study of 50 Japanese patients, Suzuki et al. found that levels of serum gd-IgA1 and IgG-gd-IgA1 immune complexes were associated with the extent of hematuria and proteinuria (155). Other than this association no other studies have been performed to allow evaluation of whether measurement of anti-gd-IgA1–gd-IgA1 complexes could be used as a biomarker in IgAN.

### Soluble CD89 (an Alternative HIT 3)

#### Biological Plausibility

CD89 (myeloid FcαR1-receptor) is a transmembrane glycoprotein found mainly on myeloid cells, which functions as a receptor for the Fc component of human IgA (156). Interaction with IgA can induce shedding of CD89 from myeloid cell surfaces, generating a soluble form of the receptor (sCD89) (157). It has been hypothesized that sCD89 may play a role in circulating immune complex formation in IgAN (158). In mouse models it has been demonstrated that sCD89-IgA complexes can deposit in the mesangium and through interaction with the transferrin receptor (CD71) and tissue transglutaminase-2 (TG-2) produce an IgAN-like disease with haematoproteinuria and kidney function decline (159). Soluble CD89 has been detected in both the urine and serum of patients with IgAN (47, 160, 161).

#### Diagnosis

In a small French cohort of 30 patients, Launay et al. found levels of sCD89 in PEG (polyethylene glycol) precipitated serum were elevated compared to levels in 30 healthy subjects and 45 disease controls (162). However, there was considerable variation in the sCD89 levels in the IgAN cohort, preventing its development as a diagnostic test. Furthermore, a Dutch group subsequently reported a lack of specificity, with sCD89 being detected in equal amounts in IgAN and healthy subjects (163).

#### Prognosis

A low serum sCD89-IgA immune complex level at the time of diagnosis (presumed due to consumption in the kidney) has been associated with a worse prognosis in a cohort of Swedish IgAN patients (160). This could not, however, be replicated in a patient cohort from Korea (160, 161). In a multi-center study of 160 Caucasian patients with IgAN or IgA vasculitis that measured urinary levels of sCD89 and TG-2, Moresco et al. found that both sCD89 and TG-2 levels were lower in patients with active disease compared to those in remission. However, both markers correlated closely with proteinuria suggesting that for prognostication there is little added benefit of measuring sCD89 and TG2 in addition to proteinuria (47).

#### Treatment Selection and Monitoring Response to Treatment

There is no data to support the use of sCD89 or sCD89-IgA immune complexes in guiding therapy decisions in IgAN.

### Inflammation, Remodeling, and Fibrosis in the Kidney (HIT 4)

Current evaluation of the kidney biopsy in IgAN includes light microscopy of normally 4–5 differently stained kidney sections, immunostaining for a limited panel of immune components and, when available, electron microscopy, alongside scoring of the PAS-stained section to generate the Oxford MEST-C score. The techniques employed have changed little over the past 50 years. Apart from the Oxford score, there have been many studies that have reported specific features in the routine evaluation of the biopsy that are part of the immunological response and may aid prognostication in IgAN including intensity and distribution of glomerular IgA immune complexes both using immunostaining and electron microscopy, co-deposition of IgG, and intensity of Complement C3 deposition. These studies have not been validated in large populations and currently it is unclear whether any of these features add value above the traditional biomarkers used in clinical practice. Future work mirroring the methodologies employed by the International IgAN Network and Renal Pathology Society to formulate the Oxford Classification may identify and validate features, currently routinely assessed in all IgAN kidney biopsies, that could add value in risk prediction and treatment decision making. In addition, with the introduction of machine learning in nephropathology it may be possible in the future to extract novel morphological biomarkers from routinely stained kidney sections that can contribute to improving prognostic accuracy of the MEST-C score and inform treatment decisions (164).

It will not be surprising that there are many hundreds of individual, non-validated, studies reporting over or under expression of specific protein biomarkers in small cohort studies in kidney biopsies in IgAN and it is impossible to cover all of these in this review. It is also impossible to comment on whether measurement of any of these proteins adds value to prognostication and/or treatment decisions in IgAN. However, as the number of novel and repurposed immunomodulatory therapies being evaluated in IgAN steadily grows, the need to be able identify biomarkers of specific intrarenal pathomechanisms is becoming increasingly important as this will ultimately aid appropriate treatment selection. In our view the immediate priority for biomarker development is to identify biomarkers that will aid with the selection of patients and monitoring of response to the increasing number of new drugs that inhibit different components of the complement system. A number of which, are currently being evaluated in IgAN, including Cemdisiran (NCT03841448: a small interfering RNA that inhibits hepatic C5 synthesis), LNP023 (NCT03373461: small molecule factor B inhibitor), IONIS-FB-LRx (NCT04014335: anti-sense inhibitor of factor B), and Narsoplimab (NCT03608033: human monoclonal antibody directed against mannose-binding lectin-associated serine protease 2).

### The Complement System: A Major Driver of Inflammation and Remodeling in IgAN

The complement system comprises more than 30 proteins and protein fragments that are part of the innate arm of the immune system and function as a cascade to amplify local inflammatory responses to foreign pathogens, or host injury signals. The common pathway of the complement system can be triggered by one of three routes; the lectin pathway, the alternative pathway or the classical pathway (165).

#### Biological Plausibility

Complement components C3 and properdin are present in 80%–90% of all kidney biopsies in IgAN. Complement components C4 or C4d, mannose-binding lectin (MBL), and the terminal complement complex (C5b–C9) are frequently detected, whereas the typical absence of C1q suggests that the classical pathway is not activated. By contrast, there is an increasing body of evidence supporting a role for the lectin pathway activating complement in IgAN. *In vitro* studies have demonstrated that polymeric IgA purified from patients with IgAN can strongly activate the lectin complement pathway and this may be amplified further by the *O*-glycosylation changes that define gd-IgA1 (166, 167). This activation is likely amplified rapidly by the alternative pathway, which is dysregulated in many IgAN cases associated with mutations in alternative pathway genes.

Importantly, when assessing complement activation in IgAN while changes in serum levels of complement components including C3, C4, CFHR5 and mannose binding lectin (MBL) have been variably reported these changes are inconsistent, not validated and at present clinically uninformative, making evaluation of complement activation *in situ* in the kidney and in the urine an essential focus for future biomarker studies in IgAN. There have been few published studies of complement component excretion in the urine and in our view this is a missed opportunity to identify novel biomarkers of kidney complement activation and response to therapy, which we are sure will be the focus of discovery and validation studies by those investigators currently examining complement therapies in IgAN.

#### Prognosis

There are multiple lines of evidence to support an association between the extent of glomerular complement activation and severity of disease in IgAN, although at present there is no standardized and validated “complement staining panel” for routine use in clinical practice in IgAN. Glomerular C3 deposition and presence of the terminal complement complex (C5b-9) have been correlated with severity of histological damage and reported as independent risk factors for kidney function decline (168). Glomerular CFHR5 deposition, a key regulator of the alternative pathway, is similarly associated with IgAN progression (169). Likewise, glomerular deposition of MBL, a key component of the lectin pathway, is associated with more severe proteinuria and histological injury (167). Further supporting the importance of lectin pathway activation, Espinosa et al. found C4d deposition (in the absence of C1q, C4d deposition signifies lectin pathway activation) was associated with more severe histological damage, and was an independent risk factor for disease progression (170). In each of these studies immunostaining was carried out in isolation of other components of the complement pathway, and therefore, it is impossible to determine the relative contribution of lectin and alternative pathway activation to progression of IgAN. There have also been no analyses to evaluate the value of adding complement immunostaining to the IgAN risk prediction score and so we must await these analyses before we are able to conclude on the importance of *in situ* complement activation in predicting prognosis in IgAN.

#### Treatment Selection and Monitoring Response to Treatment

Ahead of using a complement-directed therapy in IgAN it would be desirable to know the extent of complement activation occurring in the kidneys and the dominant pathway driving glomerular inflammation. As already mentioned studies of complement activation in the kidney have thus far studied pathways in isolation and what we need going forward is a validated “complement panel” for staining kidney biopsies that (1) confirms complement activation, (2) assesses the amount of complement activation, and (3) determines the relative contribution of lectin and alternative pathways- both to justify the use of a complement therapy and direct therapy toward alternative pathway (e.g., LNP023), lectin pathway (e.g., Narsoplimab), or common pathway (e.g., Cemdisaran) inhibition. These studies will hopefully be undertaken in the near future.

## Moving Evaluation of the Kidney Biopsy in IgAN Into the 21^st^ Century: Multi-Omics–Based Biomarker Discovery and the Kidney Biopsy

With the development of novel molecular techniques it is now possible to generate highly detailed transcriptomic landscapes not only of whole kidney tissue but also microdissected glomerular and tubulointerstitial compartments. Techniques available include bulk RNA-sequencing (RNA-seq), single nuclei RNA sequencing (snRNA-seq), and single nuclei Assay for Transposase-Accessible Chromatin sequencing (snATAC-seq). From this data, it is possible using bioinformatic tools to estimate both the composition of cell types within the whole biopsy and the expression of genes in those cell types. Early transcriptomic analysis of kidney tissue in IgAN has focussed on bulk RNA-seq and has identified specific transcriptomic signatures associated with specific histopathological lesions (171). These studies are at an early stage but it is likely that over the next decade transcriptomic analysis of the kidney biopsy in IgAN will provide important prognostic information and aid treatment decisions.

In addition to conventional transcriptomics, there have also been a small number of miRnomic studies performed in kidney tissue in IgAN. MicroRNAs (miRs) are short, non-coding oligonucleotides that regulate gene expression by disrupting translation. Since their discovery in 1993 multiple pathophysiological roles of miRs have been reported in a wide variety of conditions including IgAN (78). A small number of reports have identified miRs that are associated with components of the “four hit” hypothesis, including generation of gd-IgA1 (172, 173) and inflammation, remodeling and fibrosis in the kidney. A cluster of miRs (miRs 21-5p, 155, 199a-5p, 205, and 214-3p) have been associated with development of fibrosis and interstitial scarring in IgAN (174–177); miRs 21-5p, 214-3p, and 199a-5p are associated with kidney function decline, while others are associated specifically with mesangial inflammation (178, 179) and the modulation of endocapillary hypercellularity (180). In parallel with changes in miR expression in the kidney, changes in urinary miR excretion have also been reported in IgAN. While all of these studies are small and currently not validated we feel there is a potential for future development of miR biomarkers in IgAN.

In addition to molecular techniques, proof of concept studies are now emerging for advanced proteomic and metabolomic analysis of the kidney biopsy and urine to identify novel biomarkers, although these studies are very much in their infancy and their findings are unlikely to be integrated into clinical practice for the foreseeable future (181–184).

## Emerging Modifiers of the “Four Hits” and Their Potential Roles as Biomarkers in IgAN

### Genomic Biomarkers in IgAN

A number of genome wide association studies (GWAS) have identified risk alleles associated both with the development of IgAN and the risk of progression to ESKD. A number of these risk alleles are associated with genes directly involved in modulating the immune response. With the advent of next-generation sequencing (NGS), whole exome sequencing (WES), and whole genome sequencing (WGS) the depth of genomic knowledge of IgAN will increase exponentially over the next decade. To date integrating genomic data into risk scores for prognostication and treatment decision making has not been performed in IgAN. As an example of the possible utility of the currently available genomic data we generated an IgAN-Genetic Risk Score (GRS) using 14 single-nucleotide polymorphisms (SNPs) drawn from the largest European GWAS and calculated the IgAN-GRS in 464 biopsy proven IgAN Caucasian cases from the UK Glomerulonephritis DNA Bank and in 379 767 Europeans in the United Kingdom BioBank (UKBB) (185). We used the mean of IgAN-GRS to calculate the proportion of potential IgAN cases in subjects with hematuria and other non-specific kidney phenotypes in the UKBB. We estimated that IgAN accounted for 19% of hematuria cases and 28% of cases with hematuria, hypertension, and microalbuminuria in the UKBB. In this study, we used an IgAN-GRS to estimate the prevalence of IgAN contributing to common phenotypes that would not normally be biopsied. Further work is needed to assess if an IgAN-GRS may be useful for individual diagnosis and aid prognostication in IgAN.

### Microbiomic Biomarkers in IgAN

There has been increasing interest in the role of the microbiome in IgAN, in particular as part of the gut-kidney axis and as a stimulus for gd-IgA1 synthesis and release into the circulation. IgAN may flare during mucosal inflammation and infection (186) and development of glomerular IgA deposits in at least two murine models of IgAN is dependent upon the presence of commensal gut bacteria (162, 187). Furthermore, GWAS have identified risk alleles associated with genes involved in maintaining the integrity of the gut mucosal barrier in IgAN (188). Reduced gut microbial diversity has been reported to be associated with progressive IgAN compared to non-progressors and healthy subjects (189). Exploration of the gut microbiome to identify non-invasive biomarkers is therefore of great interest, although again we are at the start of the biomarker discovery and validation journey. With the potential for the introduction of a targeted-release formulation of budesonide (NEFECON®) for the treatment of IgAN, which is designed to deliver budesonide to the Peyer’s patches in the terminal ileum, identifying biomarkers that help us monitor the impact of gut-directed therapies is likely to be important in monitoring local response to therapy.

## Biomarkers to Predict Recurrence of IgAN in the Kidney Transplant

In those patients who develop ESKD, transplantation is the preferred method of kidney replacement therapy. Recurrence of IgAN in the transplant is a significant issue, occurring in up to 60% of patients (190–193). Predicting which patients are most likely to develop recurrent disease would facilitate more effective pre-transplantation counseling and may in the future allow a personalized approach to the use of pre-emptive treatments, when these therapies become available.

In a small study of 38 transplant recipients Berthelot et al. measured IgA–sCD89 complexes, gd-IgA1 and IgG-anti-IgA antibodies pre-transplant and correlated these levels to risk of IgAN recurrence (194). Pre-transplant gd-IgA1 and IgG-anti-IgA antibody levels were higher and IgA-sCD89 complexes lower in patients who went on to develop disease recurrence compared to those who did not develop recurrent disease. While these results are of interest, a number of confounding factors that may have influenced risk of recurrence were identified including use of basiliximab or anti-thymocyte globulin (ATG) induction regimens.

## Conclusion

We are in desperate need of validated IgAN-specific biomarkers to support treatment decision making and prognostication. At present, we heavily rely on traditional, largely non-specific, biomarkers of generic kidney disease to guide our care of patients with IgAN. While there have been many studies reporting novel biomarkers in IgAN, at present, none of these have translated into standard clinical care, largely due to the small nature of individual studies and the almost uniform absence of properly designed validation studies. If all of the new therapies currently being evaluated in clinical trials, which include endothelin receptor antagonists, B cell directed therapies, mucosal steroids, and complement inhibitors, are shown to be safe and effective clinicians are going to need robust biomarkers to both help guide their choice of treatment and monitor response to therapy in their patients with IgAN. Our hope is that the bioresources generated as part of these large global trials will facilitate robust biomarker discovery and validation studies, which in the fullness of time may result in new biomarkers translating into the clinic.

## Author Contributions

ST authored the CD89 section of this work. SS authored the section on complement. CC authored the section on the microbiome. HS and JB contributed equally to the remainder of the manuscript. HS, CC, and JB were involved in editing the manuscript and JB was the supervising author. All authors contributed to the article and approved the submitted version.

## Funding

HS is a Kidney Research UK funded clinical research training fellow. CC received research grants from GlaskoSmithKline and Retrophin. The funders were not involved in the study design, collection, analysis, interpretation of data, the writing of this article or the decision to submit it for publication.

## Conflict of Interest

The authors declare that the research was conducted in the absence of any commercial or financial relationships that could be construed as a potential conflict of interest.

The handling editor declared a past co-authorship with one of the authors JB.
